# The sealing ability of different endodontic biomaterials as an intra-orifice barrier: evaluation with high-performance liquid chromatography

**DOI:** 10.2340/biid.v11.41069

**Published:** 2024-07-24

**Authors:** Sahar Shakouei, Negin Ghasemi, Parvin Zakeri-Milani, Afsaneh Shahali, Mahdieh Alipour

**Affiliations:** a Department of Endodontics, Dental Faculty, Tabriz University of Medical Sciences, Tabriz, Iran; b Drug Applied Research Center and Faculty of Pharmacy, Tabriz University of Medical Sciences, Tabriz, Iran; c Dental Faculty, Tabriz University of Medical Sciences, Tabriz, Iran; d Dental and Periodontal Research Center, Faculty of Dentistry, Tabriz University of Medical Sciences, Tabriz, Iran; e Stem Cell Research Center, Tabriz University of Medical Sciences, Tabriz, Iran

**Keywords:** Biodentine, CEM cement, bleaching, high-performance liquid chromatography (HPLC), MTA

## Abstract

**Objective:**

This study evaluated the sealing ability of different biomaterials as intra-orifice barriers in the internal bleaching of discolored teeth with the walking bleaching technique. The release of hydroxyl ions from the bleaching materials can cause cervical root resorption, making it necessary to use intra-orifice barrier materials to prevent this issue.

**Materials and methods:**

In the current study, the high-performance liquid chromatography (HPLC) method was used to measure the released hydroxyl ions. The study included 90 single-rooted and single-canal premolars, which were divided into four groups based on the intra-orifice barrier materials used (mineral trioxide aggregate [MTA], calcium-enriched mixture [CEM], Biodentine, and MTA+PG) and the type of bleaching material (sodium perborate + water or sodium perborate + hydrogen peroxide 30%). Two control groups were also considered in this study: a positive control group, where sodium perborate and hydrogen peroxide were placed inside the pulp chamber without any intra-orifice barriers; and a negative control group, where no bleaching agent or surgical obstruction was used, and the root surface was covered with wax up to the cemento-enamel junction (CEJ) level.

**Results:**

The results showed that there was a significant difference in the concentration of hydroxyl ions released among the studied groups. The amount of hydroxyl ion released was highest in the positive control group and lowest in the CEM group. Among the intra-orifice barrier materials used, CEM cement was found to be the most appropriate material for use in the step-by-step internal bleaching method.

**Conclusions:**

The study highlights the importance of using appropriate intra-orifice barrier materials to prevent root cervical resorption in internal bleaching procedures.

## Introduction

Discoloration of teeth after root canal treatment is a common problem that can be caused by various reasons, such as the presence of gutta-percha and sealer or remaining pulp tissue in the area of the pulp horns. One of the effective methods to treat this issue is internal tooth bleaching using a walking bleaching technique [1]. This method involves placing an intra-orifice barrier in the coronal area of the canal and then applying a bleaching material. Although sodium perborate with water has been proven to be effective, it requires frequent replacement of the substance and multiple treatment sessions [2, 3]. In contrast, the combination of sodium perborate and hydrogen peroxide requires fewer treatment sessions [4].

However, the use of hydrogen peroxide as a bleaching material can cause cervical root resorption due to the diffusion of hydroxyl ions from the material to the periodontal tissues [5]. To prevent diffusion, a series of materials can be placed as a barrier in the coronal area of the canal, including Cavit, glass ionomer cement, and MTA [3]. Although MTA has many desirable properties, its handling is difficult, and it has a long setting time. Therefore, other biomaterials, such as calcium-enriched mixture (CEM) and Biodentine, have been introduced in the endo field and have similar applications to MTA but better properties [6–8].

One way to improve the properties of MTA is to add compounds to its composition, such as propylene glycol, which has been shown to improve its consistency, handling, and bond strength without causing a negative biological response [9]. However, there is no published study on the sealing ability of these materials as an intra-orifice barrier.

Previous studies have used qualitative or imprecise quantitative methods to investigate sealing ability, such as dye leakage, saliva leakage, protein leakage, and microbial leakage [9–12]. In contrast, the high-performance liquid chromatography (HPLC) method is a precise and quantitative method that has recently received attention in studies [13]. The HPLC method uses terephthalic acid, which reacts with hydroxyl ions to create a substance with fluorescence properties called hydroxy terephthalic acid. This method was used in this study to investigate the flooding ability of different endo biomaterials as an intra-orifice barrier in bleaching.

The purpose of this study was to investigate the sealing ability of different endo biomaterials, including Biodentine, MTA+PG (propylene glycol), MTA, and CEM, as an intra-orifice barrier in the internal bleaching method, which was done by estimating the amount of hydroxyl ions released by the HPLC method. This study aimed to provide valuable information for dental professionals to choose the most suitable material as an intra-orifice barrier in internal tooth bleaching procedures.

## Methods and materials

For this study, 90 extracted mandibular premolars were used. These teeth were obtained from patients after written informed consent. This research has been approved by Tabriz University of Medical Science (approval code: IR.TBZMED.REC.1400.403).

After the preparation of the access cavity, a size 15 k-file (Dentsply Maillefer, Ballaigues, Switzerland) was placed in the canal to the apex. Three millimeters were subtracted from this length to determine the working length. The canal was prepared using the RaCe rotary system (FKG Dentaire, La Chaux-de-Fonds, Switzerland) as follows: a size 40 rotary file was used at 10% for the coronal third, a size 35 file was used at 8% for the middle third, and a size 30 file was used at 6% until the working length. A 2.5% hypochlorite solution was used for irrigation during the procedure. Finally, the smear layer was removed using a 5.25% sodium hypochlorite solution for 3 min, followed by 17% EDTA for 3 min. Normal saline was used as the final rinse. The canal was then obturated using the lateral compaction technique with gutta-percha and AH-26 sealer (Dentsply, Detrey, Konstanz, Germany). After that, using a heat carrier, 3 millimeters of gutta-percha from the sub-CEJ cervical area were removed to place the intra-orifice barrier. The list of materials is provided in Table 1.

**Table 1:** List of materials used in the study.

Name of product	Company
Gutta-Percha	Dentsply, Detrey, Konstanz, Germany
AH-26 sealer	Dentsply, Detrey, Konstanz, Germany
MTA	Angelus Dental Industry Products, Londrina, Brazil
CEM	YektazistDandan, Tehran, Iran
Biodentine	Septodont, St. Maur-des-Fossés, France
Propylene Glycol (PG)	Merk, Darmstadt, Germany

The premolars were then assigned to the following groups based on their intra-orifice barrier:

MTA (Angelus Dental Industry Products, Londrina, Brazil), CEM (YektazistDandan, Tehran, Iran), Biodentine (Septodont, St. Maur-des-Fossés, France), MTA+ PG (Merk, Darmstadt, Germany) as well as a positive and a negative control group described below.

All mentioned biomaterials were mixed according to the manufacturer’s instructions as follows:

MTA: The ratio of powder to liquid was 3 to 1. CEM: Liquid was gradually added to the powder until a thick consistency was achieved. Biodentin: 5 drops of the kit liquid were added to the capsule, and the mixing was done using an amalgamator for 30 s.

MTA+PG (propylene glycol): According to previous studies, the best percentage of adding propylene glycol to MTA is 20% volume [9]. In fact, 4 cc of MTA liquid was mixed with 1 cc of propylene glycol, which was measured with an insulin syringe. The ratio of MTA powder to liquid was 3 to 1, and the mixing was done manually.

After placing the material in cervical one-third of the root canal using an MTA carrier and condensing it with an appropriate condenser, a moist cotton pellet was placed in the pulp chamber by one person in all samples. The samples were kept in an incubator at 37°C for 24 h. After confirming the setting of the materials, the samples of each group were divided into two subgroups, A and B based on the bleaching material used:

Subgroup A: Sodium perborate was mixed with water in a 2:1 (ml/g) ratio and placed inside the pulp chamber. The treated teeth were sealed with Cavit.

Subgroup B: Sodium perborate was mixed with 30% hydrogen peroxide in a similar ratio as the previous subgroup and placed inside the pulp chamber. The treated teeth were sealed with Cavit.

For positive and negative control groups, these treatments were considered:

Positive control: Sodium perborate and hydrogen peroxide were placed inside the pulp chamber without any intra-orifice barriers.

Negative control: No bleaching agent or surgical obstruction was used, and the root surface was covered with wax up to the CEJ level.

To measure the amount of hydroxyl ions leaked from each tooth, 1 cc of terephthalic acid was poured into a test tube, and the tooth was placed in the tube and lowered into the solution until at 3 mm apical of CEJ region. The samples were incubated for 48 h at 37°C.

HPLC was used to measure and determine the amount of hydroxyl ions. In HPLC, Terephthalic acid was used, which in the presence of hydroxide ion generates a specific fluorescent substance called hydroxy terephthalic acid. This substance is detected by the instrument, and the amount of free hydroxide ion released is evaluated based on it.

For statistical analysis, after calculating the mean and standard deviation, the normality of the data distribution for the study groups was examined using the Smirnov–Kolmogorov test to determine the amount of released free hydroxyl ions. Two-way ANOVA was used to test for a significant effect of the type of intra-orifice barrier material and bleaching material on the level of hydroxyl ions, and Tukey’s post hoc test was used for pairwise comparisons of groups. Two-way ANOVA was also used for pairwise comparison of groups. A significant level of *P* < 0.05 was considered, and the statistical analysis was performed using SPSS 17 software.

## Results

**Figure 1:**
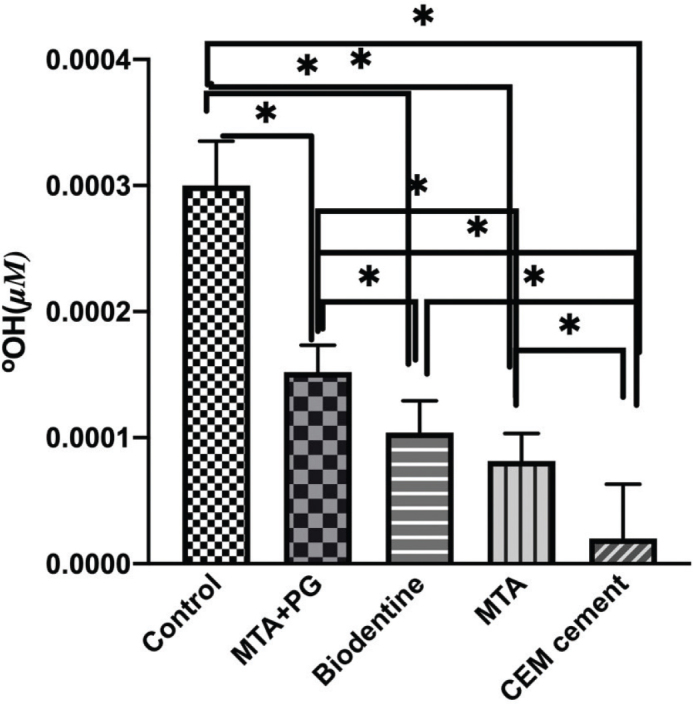
Release of hydroxyl ions in different biomaterials used as an intra-orifice barrier. CEM cement showed significantly decreased levels of hydroxyl ions compared with all other biomaterials. There were no significant differences between MTA and Biodentine. *P* < 0.05.

The concentration of released hydroxyl ions with both sodium perborate and sodium perborate was mixed with 30% hydrogen peroxide is presented in Figure 1. As observed, the highest amount of released free hydroxyl ions was in the positive control group, and the lowest amount was in the CEM group. The negative control lacked any bleaching agents, resulting in no release of hydroxyl ions. There was a statistically significant difference in the concentration of free hydroxyl ions among all studied groups except between MTA and Biodentine.

**Figure 2:**
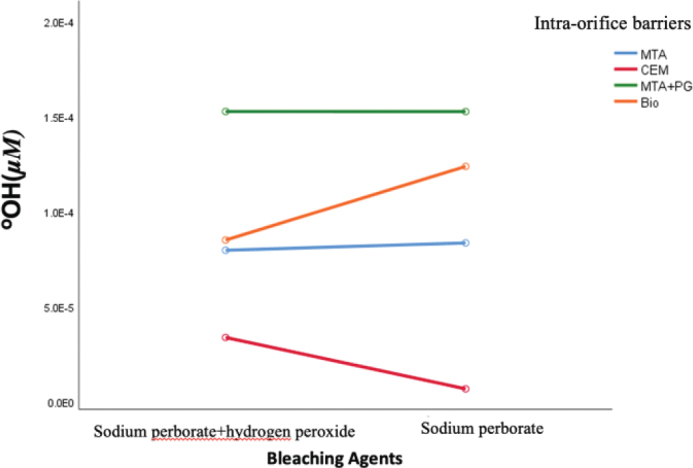
Mean concentration of hydroxyl ions in different intra-orifice barriers based on the bleaching agent. There is no significant difference in the amount of hydroxyl released between bleaching agents.

Although our results showed that the amount of hydroxyl released among intra-orifice barrier materials was statistically significant, there was no statistically significant difference in the amount of hydroxyl released between bleaching materials (*p* = 0.58). The amount of hydroxyl released in different intra-orifice barriers in the presence of two bleaching groups is shown in Figure 2.

**Figure 3:**
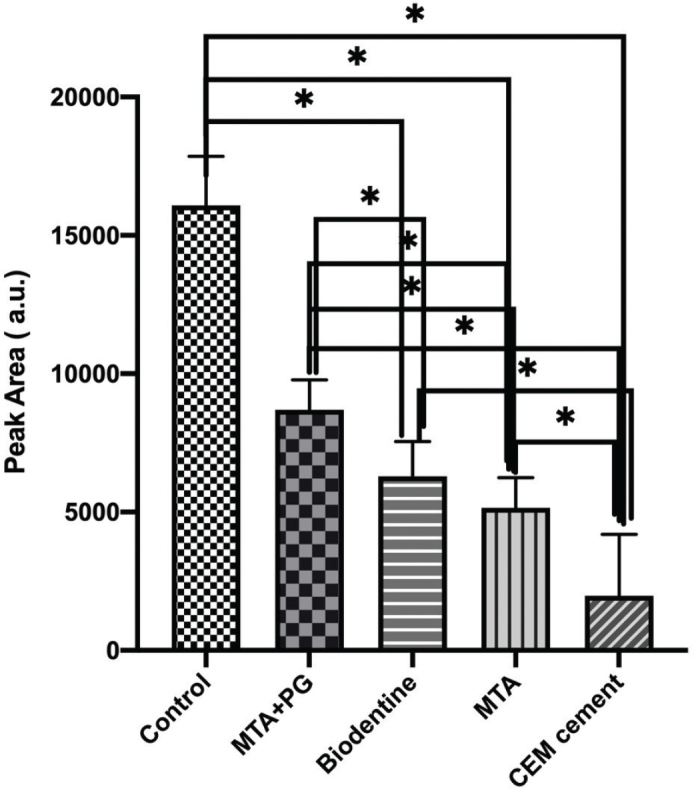
The peak area in the studied groups showed significant differences among all studied groups except between MTA and Biodentine. *P* < 0.05.

In Figure 3, the peak area of different biomaterials is presented. The results showed that the highest peak area of the released ions was observed in the positive control group, and the lowest value was observed in the CEM group. Pairwise comparisons of the studied dental materials showed statistically significant differences in mean peak area between all materials (*p* = 0.27) except between Biodentine and (*p* < 0.05).

## Discussion

The aim of this study was to evaluate the amount of hydroxyl ion leakage in the presence of different intra-orifice barriers in the internal bleaching method with a walking bleaching technique, which was performed using HPLC. The results showed that CEM had the lowest and MTA+PG the highest amount of leakage. The amount of hydroxyl ion leakage to the root surface in the presence of sodium hypochlorite with water or hydrogen peroxide did not differ between any of the intra-orifice barriers. The internal bleaching method with the walking system is one of the methods for changing the color of discolored teeth after root canal treatment. In this technique, placing the barrier material in the coronal portion of the canal is essential [1]. After placing a bleaching agent in the tooth, the pH of the root surface becomes acidic, which increases the activity of osteoclasts on the root surface and allows for cervical resorption. Therefore, it is highly desirable to place materials as a barrier that can change the pH of the environment to an alkaline state [4, 14]. Materials such as CEM, MTA, and Biodentine have this property due to their formulation containing calcium oxide, which converts to calcium hydroxide in combination with water [6, 15]. On the other hand, these materials have the ability to chemically and physically bond with dentin, which can be useful in preventing leakage [16, 17]. Among the materials used, CEM showed the best performance, which is consistent with previous studies that have shown CEM to have better or equal bonding ability to MTA [10, 18, 19]. In contrast to this study, a study using a dye penetration method to evaluate the sealing ability of CEM cement and MTA during internal bleaching of teeth showed no significant differences between the two materials [3].

Another study by Neshat, comparing the biocompatibility of three materials, Biodentine, MTA, and CEM cement, showed that the setting time of these three materials did not differ significantly [20]. In the current study, there was no significant difference between MTA and Biodentine, but the setting time of CEM cement was significantly shorter than the other two materials. A study in agreement with the current one showed that CEM cement has advantages over MTA, such as a shorter setting time, better handling, no discoloration of the tooth, and effective sealing against bacterial leakage [21]. Studies have also shown better biocompatibility of CEM and less inflammation when in contact with live cells [22, 23]. Furthermore, a greater thickness of the dentin bridge was reported below CEM compared with MTA [23]. Regarding antibacterial effects, a higher antibacterial effect of CEM was demonstrated compared to MTA [24–26]. When CEM is mixed with water-based liquid, bioactive calcium, and phosphate-rich materials are formed, which are useful for root canal sealing materials. The higher concentration of phosphorus in CEM compared to MTA showed the better sealing of CEM in the current study [27]. According to another study using dye penetration, the amount of CEM leakage was lower than that of MTA and IRM [28]. Furthermore, the sealing of CEM has been found to be better than that of MTA in both dry and moist environments [16, 29]. This is probably due to better handling, a high percentage of small particles (access to the pulp chamber), and a higher concentration of phosphates that produce hydroxyapatite when combined with calcium, resulting in better sealing between the material and the cavity wall [30].

In a study on the sealing ability of MTA-Angelus mixed with PG, bacterial leakage was better at the end of a 30-day period using the bacterial leakage method compared to mixing with water [31]. However, the results of that study are significantly different from our study. In our study, mixing MTA with propylene glycol, which is done to improve the handling of MTA, did not yield the desired result. In previous studies, push-out bond strength and compressive strength of MTA were reduced with the addition of propylene glycol [32]. Also, when PG is added to MTA, the amount of water available for hydration reaction is reduced, resulting in increased setting time [33]. Due to the increased setting time, the solubility and porosity of MTA increased, and as a result, the sealing ability and mechanical strength of MTA decreased [34, 35]. Additionally, the hardness of the material decreased with the addition of PG to MTA [36, 37], which confirms the results of our study that the use of MTA mixed with water is better than mixing it with PG.

In the present study, the mixing of sodium perborate bleaching agents with water and hydrogen peroxide was performed. In another study, the release of hydroxyl ions during the use of sodium perborate with water and 30% hydrogen peroxide was compared using an HPLC device with a fluorescence detector, and it was observed that the release of hydroxyl ions during the use of sodium perborate with hydrogen peroxide 30% was higher than water [14]. Other studies have yielded similar results to previous study [14], indicating that this is an important factor that reduces the treatment time during bleaching when using sodium perborate with hydrogen peroxide 30% due to the increased release of hydroxyl ions [4, 5].

Furthermore, the method used in this study to measure the number of hydroxyl ions that penetrated the outer surface of the root was HPLC. In most previous studies, the dye penetration method was used, which is a qualitative method and has less accuracy compared to quantitative methods such as chromatography [9, 10, 14].

Two points should be considered when using silicate materials as intra-orifice barriers. The first is how hydroxyl ions act as an oxidizing substance in these materials, which has been shown to reduce the bond strength of MTA.

A second issue is the risk of color change caused by intra-orifice barrier materials. It has been found that the color change induced by Biodentine and CEM does not differ, and that the color change induced by MTA is more pronounced [38, 39].

In conclusion, considering the sealing ability evaluated in this study and the results of previous studies regarding the color change, it seems that CEM is more suitable material for intra-orifice barrier functions.

## Data Availability

The datasets used and/or analyzed during the current study are available from the corresponding author on reasonable request.
